# The Genome Sequence of the Delicate moth, *Mythimna vitellina* (Hübner, [1808])

**DOI:** 10.12688/wellcomeopenres.22620.1

**Published:** 2024-07-29

**Authors:** Mark Sterling, David C. Lees, Natural History Museum Genome Acquisition Lab, Darwin Tree of Life Barcoding collective, Wellcome Sanger Institute Tree of Life Management, Samples and Laboratory team, Wellcome Sanger Institute Scientific Operations: Sequencing Operations, Wellcome Sanger Institute Tree of Life Core Informatics team, Tree of Life Core Informatics collective, Darwin Tree of Life Consortium

**Affiliations:** 1 Natural History Museum, London, England, UK

**Keywords:** Mythimna vitellina, the Delicate moth, genome sequence, chromosomal, Lepidoptera

## Abstract

We present a genome assembly from an individual male *Mythimna vitellina* (the Delicate; Arthropoda; Insecta; Lepidoptera; Noctuidae). The genome sequence is 726.7 megabases in span. Most of the assembly is scaffolded into 31 chromosomal pseudomolecules, including the Z sex chromosome. The mitochondrial genome has also been assembled and is 15.43 kilobases in length. Gene annotation of this assembly on Ensembl identified 18,228 protein coding genes.

## Species taxonomy

Eukaryota; Opisthokonta; Metazoa; Eumetazoa; Bilateria; Protostomia; Ecdysozoa; Panarthropoda; Arthropoda; Mandibulata; Pancrustacea; Hexapoda; Insecta; Dicondylia; Pterygota; Neoptera; Endopterygota; Amphiesmenoptera; Lepidoptera; Glossata; Neolepidoptera; Heteroneura; Ditrysia; Obtectomera; Noctuoidea; Noctuidae; Hadeninae; Leucanini; *Mythimna*; *Mythimna vitellina* (Hübner, [1808]) (NCBI:txid987991)

## Background

*Mythimna vitellina* (Hübner, [1808]), the Delicate, is a noctuid moth with a wingspan of 36–43 mm in the UK, which belongs to a group commonly known as Wainscot moths. Its forewing is typically pale yellowish ochreous but varies from pale straw to rich orange-red and is often flushed with rufous colour. It has three narrow, undulating, darker fasciae. Both the orbicular and reniform stigmata are slightly darker than the ground colour. The hindwing is pale greyish white, suffused darker on the veins, especially in the female ( Bretherton *et al.*, 1979). *M. vitellina* is a distinctive species with a characteristic wing pattern and yellow to brick coloured forewings (the derivation of *vitellina* is vitellus, which is Latin for egg yolk), both of which are unusual for Wainscot moths.

The species was first recorded in the UK as captured at sugar by Henry Cooke, in his own garden, at Brighton, Sussex ( Newman, 1856). In the last half of the nineteenth and the twentieth century it was a regular, if uncommon, immigrant species, recorded principally in south-western England, although occasionally, as in 1992 when over 7,000 were recorded, it was found in substantial numbers. In the last 25 years it has become increasingly common in south-western England and in other southern seaboard counties. The early stages are seldom encountered in the wild, and it is difficult to be certain about the extent to which this is now a resident species in the UK, but although its numbers are highest during periods of migrant activity, its presence in south-west England, when there is no migrant activity, indicates that it breeds in the UK during the summer, and that its early stages are now capable of surviving the winter in suitable coastal areas. Historically, most UK specimens were found in September and October, but an earlier generation is now increasingly being found in June.

*M. vitellina* is a widely distributed species in Europe (a migrant towards the north of its range including Southern Scandinavia and north-western Russia) which is also found, as a migrant or resident, in Morocco, Turkey, Iran, Uzbekistan, Kyrgyzstan, North Pakistan and the European part of Russia and east of the Caspian Sea ( GBIF Secretariat, 2024).

The larva feeds on various grasses, especially soft-bladed species ( *Poa* L. (1753), *Lolium* L., *Brachypodium* P. Beauv. 1812, *Dactylis* L. etc.). It has been regarded as a pest of maize. It is a bivoltine species in most parts of its distribution, where adults can be found from May to July, and from August to November ( Hacker *et al.*, 2002).

Morphologically, *M. vitellina* belongs to a small species group, together with *M. thomasi* Hacker, Hreblay & Plante, 1993 and *M. daemona* Hreblay and Legrain, 1996, which are distinguishable from the taxa of other groups by their forewing pattern and the configuration of the genitalia of both sexes ( Hacker *et al.*, 2002).

Genetically, *M. vitellina* belongs to a single BIN cluster on BOLD (BOLD:AAE0041) which shows within it a modest 0.45% average and 1.12% maximum divergence ( *n* = 28); the closest BIN is *Leucania venalba* (Moore, 1867) (BOLD:AAB9371), which is at least 5.24% distant, and from a similar distance to various species of *Mythimna* Ochsenheimer, 1816 and *Leucania* Ochsenheimer, 1816. The mitogenome (OX438715.1) from the genome assembly has an identical haplotype in its COI-5P region to many DNA barcodes found in Europe. *M. vitellina* has been included in the subgenus *Mythimna* ( Hacker *et al.*, 2002).

The genome presented here will be useful for phylogenetic work clarifying the relationships of *Mythimna* and *Leucania*, and also for genetic comparison with pest species of related armyworms.

## Genome sequence report

**Figure 1.**
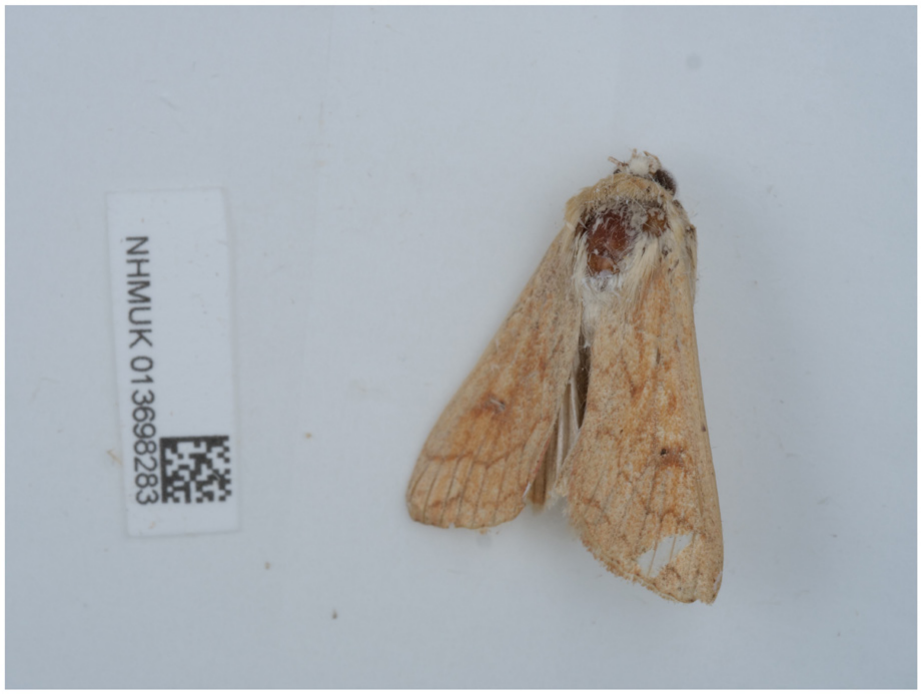
Photograph of the *Mythimna vitellina* (ilMytVite1) specimen used for genome sequencing.

The genome was sequenced from an adult male *Mythimna vitellina* ( Figure 1) collected from Sandwich Bay Bird Observatory, England, UK (51.27, 1.37). A total of 38-fold coverage in Pacific Biosciences single-molecule HiFi long reads was generated. Primary assembly contigs were scaffolded with chromosome conformation Hi-C data. Manual assembly curation corrected 10 missing joins or mis-joins and removed three haplotypic duplications, reducing the assembly length by 0.76%.

**Figure 2.**
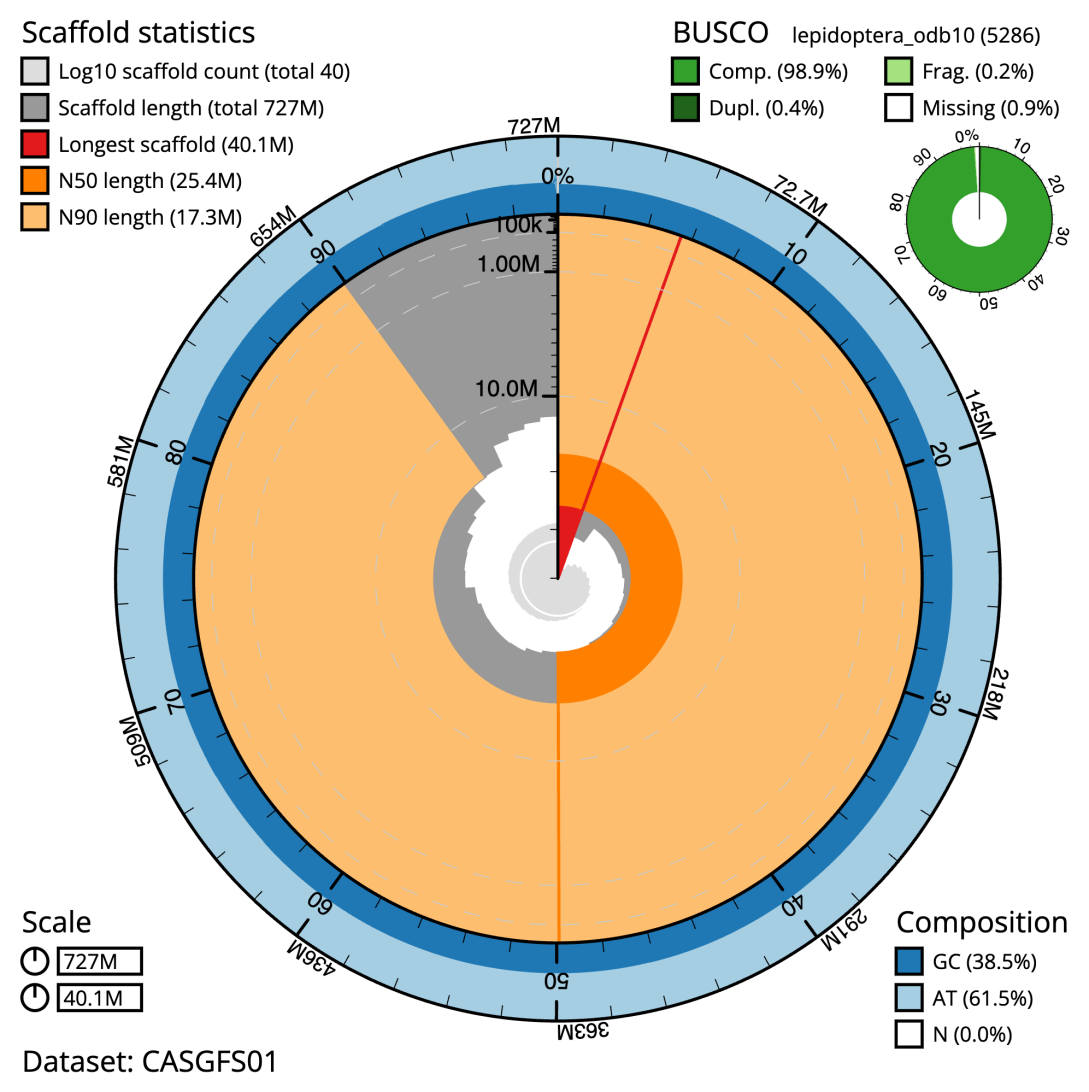
Genome assembly of *Mythimna vitellina*, ilMytVite1.1: metrics. The BlobToolKit snail plot shows N50 metrics and BUSCO gene completeness. The main plot is divided into 1,000 size-ordered bins around the circumference with each bin representing 0.1% of the 726,695,790 bp assembly. The distribution of scaffold lengths is shown in dark grey with the plot radius scaled to the longest scaffold present in the assembly (40,077,415 bp, shown in red). Orange and pale-orange arcs show the N50 and N90 scaffold lengths (25,372,425 and 17,321,795 bp), respectively. The pale grey spiral shows the cumulative scaffold count on a log scale with white scale lines showing successive orders of magnitude. The blue and pale-blue area around the outside of the plot shows the distribution of GC, AT and N percentages in the same bins as the inner plot. A summary of complete, fragmented, duplicated and missing BUSCO genes in the lepidoptera_odb10 set is shown in the top right. An interactive version of this figure is available at https://blobtoolkit.genomehubs.org/view/CASGFS01/dataset/CASGFS01/snail.

**Figure 3.**
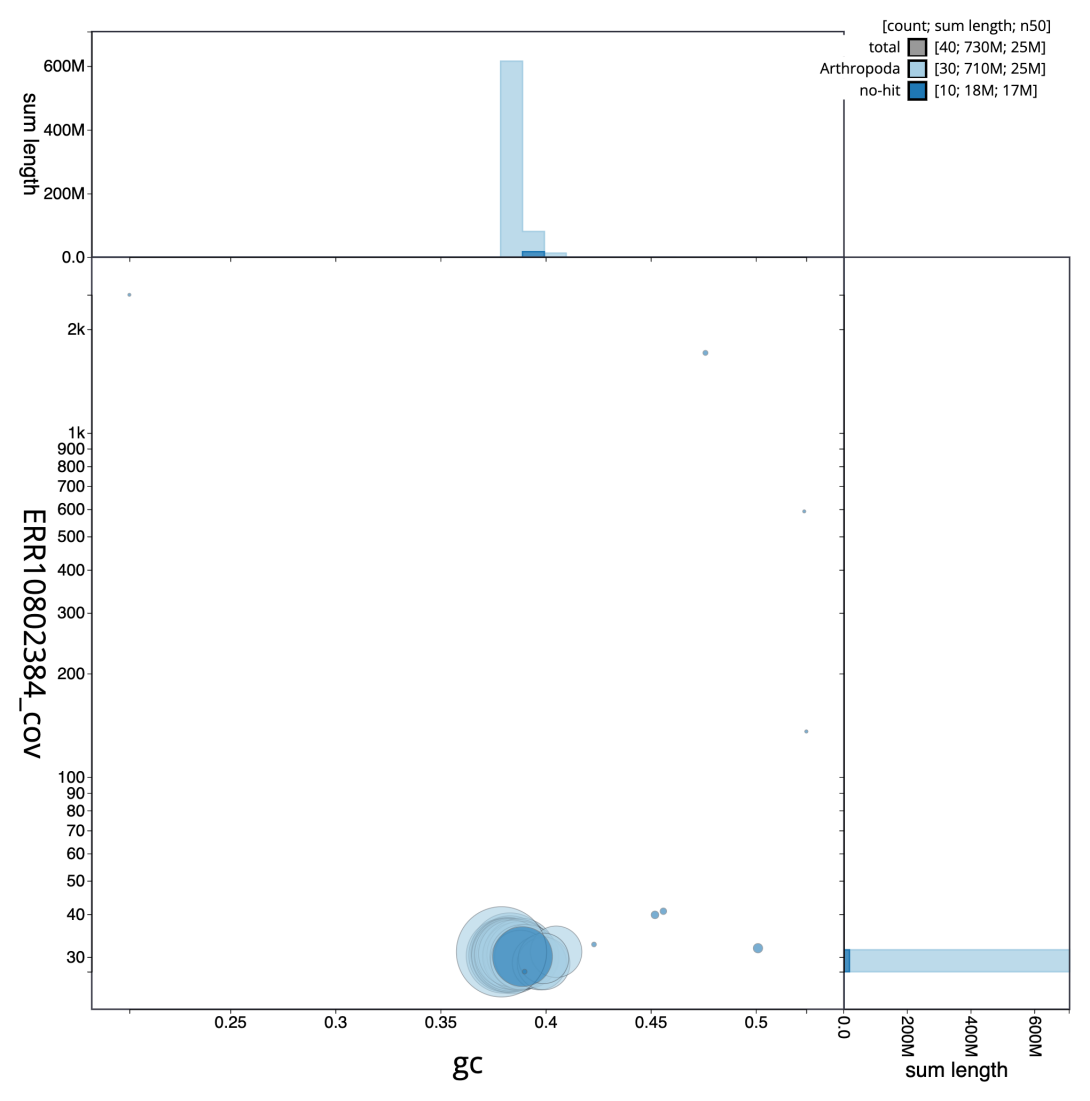
Genome assembly of *Mythimna vitellina*, ilMytVite1.1: BlobToolKit GC-coverage plot. Sequences are coloured by phylum. Circles are sized in proportion to sequence length. Histograms show the distribution of sequence length sum along each axis. An interactive version of this figure is available at https://blobtoolkit.genomehubs.org/view/CASGFS01/dataset/CASGFS01/blob.

**Figure 4.**
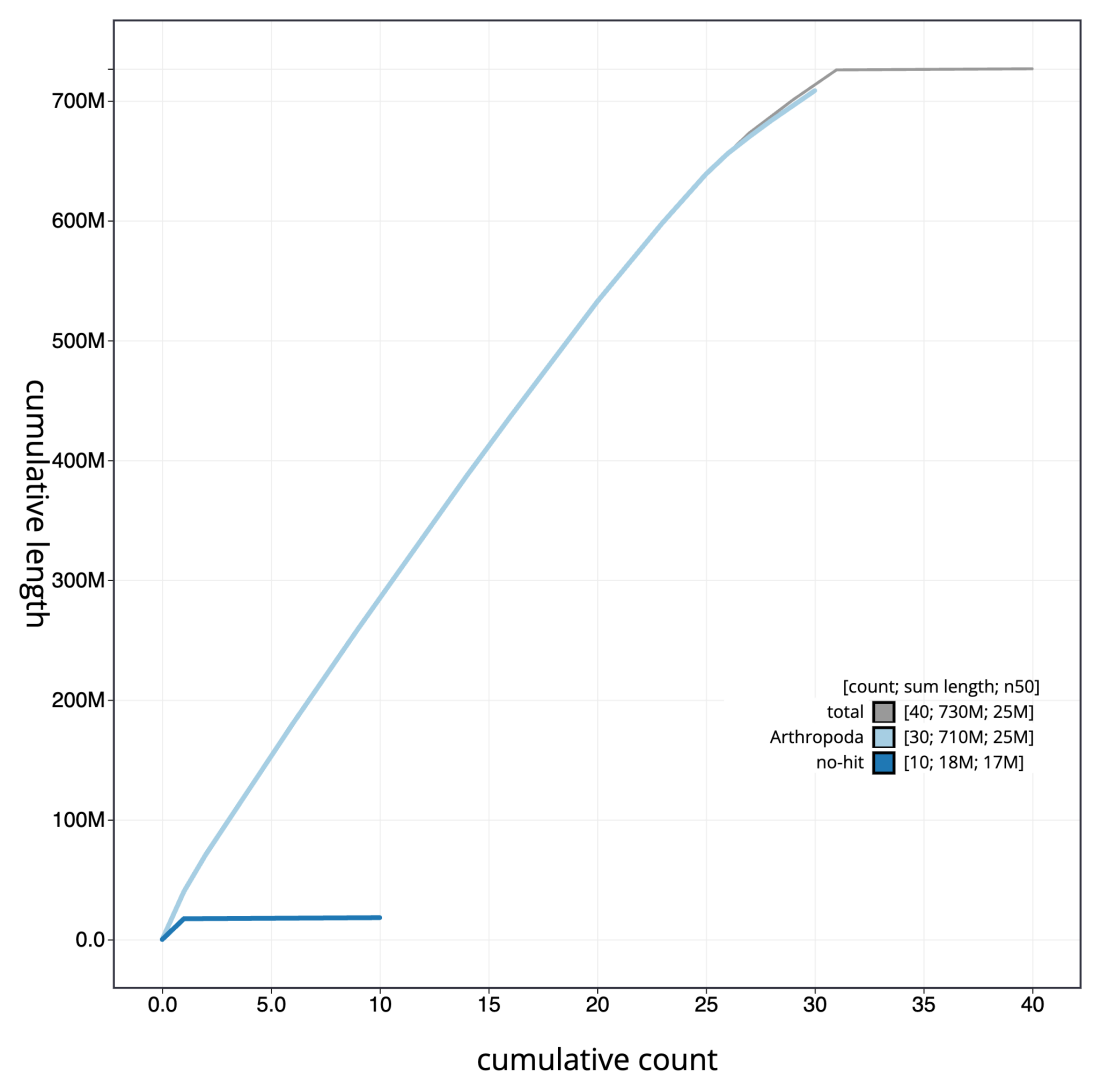
Genome assembly of *Mythimna vitellina*, ilMytVite1.1: BlobToolKit cumulative sequence plot. The grey line shows cumulative length for all sequences. Coloured lines show cumulative lengths of sequences assigned to each phylum using the buscogenes taxrule. An interactive version of this figure is available at https://blobtoolkit.genomehubs.org/view/CASGFS01/dataset/CASGFS01/cumulative.

**Figure 5.**
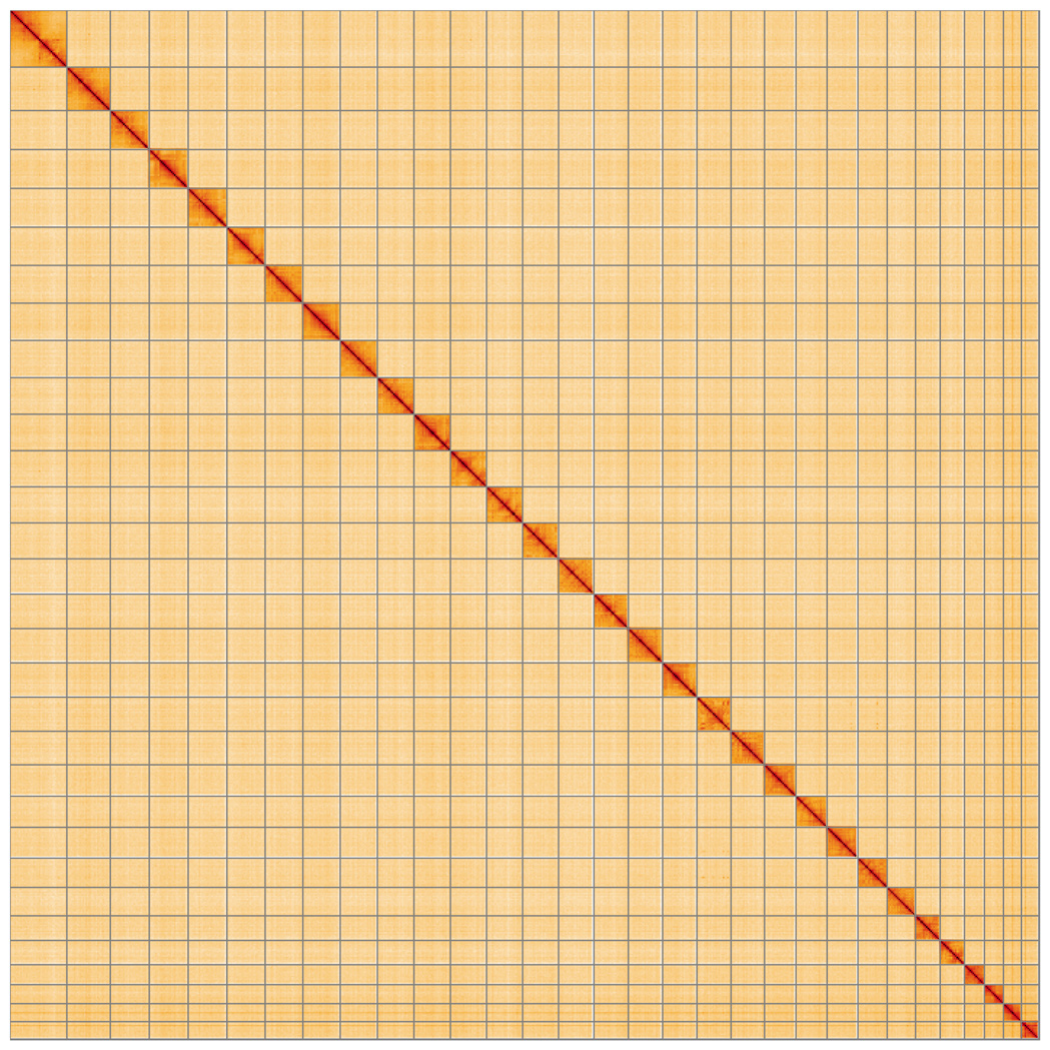
Genome assembly of *Mythimna vitellina*, ilMytVite1.1: Hi-C contact map of the ilMytVite1.1 assembly, visualised using HiGlass. Chromosomes are shown in order of size from left to right and top to bottom. An interactive version of this figure may be viewed at https://genome-note-higlass.tol.sanger.ac.uk/l/?d=Kh50TYRrS_awV5_IbJIYCA.

The final assembly has a total length of 726.7 Mb in 39 sequence scaffolds with a scaffold N50 of 25.4 Mb ( Table 1). The snail plot in Figure 2 provides a summary of the assembly statistics, while the distribution of assembly scaffolds on GC proportion and coverage is shown in Figure 3. The cumulative assembly plot in Figure 4 shows curves for subsets of scaffolds assigned to different phyla. Most (99.87%) of the assembly sequence was assigned to 31 chromosomal-level scaffolds, representing 30 autosomes and the Z sex chromosome. Chromosome-scale scaffolds confirmed by the Hi-C data are named in order of size ( Figure 5; Table 2). Chromosome Z was assigned by alignment to *Mythimna impura* (GCA_905147345.3) ( Boyes *et al.*, 2022). While not fully phased, the assembly deposited is of one haplotype. Contigs corresponding to the second haplotype have also been deposited. The mitochondrial genome was also assembled and can be found as a contig within the multifasta file of the genome submission.

**Table 1.** Genome data for *Mythimna vitellina*, ilMytVite1.1.

Project accession data		
Assembly identifier	ilMytVite1.1	
Species	*Mythimna vitellina*	
Specimen	ilMytVite1	
NCBI taxonomy ID	987991	
BioProject	PRJEB58960	
BioSample ID	Genome sequencing and Hi-C scaffolding: SAMEA111458626 RNA sequencing: SAMEA111458616	
Isolate information	ilMytVite1: head and thorax (long read sequencing and Hi-C sequencing); abdomen (RNA sequencing)	
Assembly metrics *		*Benchmark*
Consensus quality (QV)	68.9	*≥ 50*
*k*-mer completeness	100.0%	*≥ 95%*
BUSCO **	C:98.8%[S:98.4%,D:0.4%],F:0.2%,M:1.0%,n:5,286	*C ≥ 95%*
Percentage of assembly mapped to chromosomes	99.87%	*≥ 95%*
Sex chromosomes	Z	*localised homologous pairs*
Organelles	Mitochondrial genome: 15.43 kb	*complete single alleles*
Raw data accessions		
PacificBiosciences Sequel IIe	ERR10802384	
Hi-C Illumina	ERR10786031	
PolyA RNA-Seq Illumina	ERR12708739	
Genome assembly		
Assembly accession	GCA_949316375.1	
*Accession of alternate haplotype*	GCA_949316395.1	
Span (Mb)	726.7	
Number of contigs	87	
Contig N50 length (Mb)	15.2	
Number of scaffolds	39	
Scaffold N50 length (Mb)	25.4	
Longest scaffold (Mb)	40.08	
Genome annotation		
Number of protein-coding genes	18,228	
Number of gene transcripts	18,409	

* Assembly metric benchmarks are adapted from column VGP-2020 of “Table 1: Proposed standards and metrics for defining genome assembly quality” from Rhie *et al.* (2021). ** BUSCO scores based on the lepidoptera_odb10 BUSCO set using version 5.3.2. C = complete [S = single copy, D = duplicated], F = fragmented, M = missing, n = number of orthologues in comparison. A full set of BUSCO scores is available at https://blobtoolkit.genomehubs.org/view/CASGFS01/dataset/CASGFS01/busco.

**Table 2.** Chromosomal pseudomolecules in the genome assembly of *Mythimna vitellina*, ilMytVite1.

INSDC accession	Name	Length (Mb)	GC%
OX438685.1	1	30.73	38.5
OX438686.1	2	27.49	38.5
OX438687.1	3	27.42	38.5
OX438688.1	4	27.34	38.0
OX438689.1	5	26.97	38.0
OX438690.1	6	26.6	38.0
OX438691.1	7	26.33	38.5
OX438692.1	8	26.2	38.5
OX438693.1	9	25.82	38.0
OX438694.1	10	25.74	38.5
OX438695.1	11	25.46	38.5
OX438696.1	12	25.43	38.0
OX438697.1	13	25.37	38.0
OX438698.1	14	24.93	38.5
OX438699.1	15	24.3	38.5
OX438700.1	16	24.19	38.5
OX438701.1	17	24.16	38.5
OX438702.1	18	24.11	39.0
OX438703.1	19	23.64	38.5
OX438704.1	20	22.1	38.5
OX438705.1	21	21.92	39.0
OX438706.1	22	21.76	38.5
OX438707.1	23	20.68	39.0
OX438708.1	24	20.04	39.0
OX438709.1	25	17.32	39.0
OX438710.1	26	17.15	39.0
OX438711.1	27	14.13	39.5
OX438712.1	28	13.37	40.0
OX438713.1	29	12.75	40.5
OX438714.1	30	12.25	40.0
OX438684.1	Z	40.08	38.0
OX438715.1	MT	0.02	20.5

The estimated Quality Value (QV) of the final assembly is 68.9 with *k*-mer completeness of 100.0%, and the assembly has a BUSCO v5.3.2 completeness of 98.8% (single = 98.4%, duplicated = %), using the lepidoptera_odb10 reference set ( *n* = 5,286).

Metadata for specimens, barcode results, spectra estimates, sequencing runs, contaminants and pre-curation assembly statistics are given at https://links.tol.sanger.ac.uk/species/987991.

## Genome annotation report

The *Mythimna vitellina* genome assembly (GCA_949316375.1) was annotated at the European Bioinformatics Institute (EBI) on Ensembl Rapid Release. The resulting annotation includes 18,409 transcribed mRNAs from 18,228 protein-coding genes ( Table 1; https://rapid.ensembl.org/Mythimna_vitellina_GCA_949316375.1/Info/Index).

## Methods

### Sample acquisition and nucleic acid extraction

A male adult *Mythimna vitellina* (specimen ID NHMUK013698283, ToLID ilMytVite1) was hand-picked from Sandwich Bay Bird Observatory, England, UK (latitude 51.27, longitude 1.37) on 2021-09-24. The specimen was collected and identified by David Lees (Natural History Museum) and preserved by dry freezing at –80 °C.

The workflow for high molecular weight (HMW) DNA extraction at the Wellcome Sanger Institute (WSI) Tree of Life Core Laboratory includes a sequence of core procedures: sample preparation; sample homogenisation, DNA extraction, fragmentation, and clean-up. In sample preparation, the ilMytVite1 sample was weighed and dissected on dry ice ( Jay *et al.*, 2023). Tissue from the head and thorax was homogenised using a PowerMasher II tissue disruptor ( Denton *et al.*, 2023a).

HMW DNA was extracted in the WSI Scientific Operations core using the Automated MagAttract v2 protocol ( Oatley *et al.*, 2023). The DNA was sheared into an average fragment size of 12–20 kb in a Megaruptor 3 system with speed setting 31 ( Bates *et al.*, 2023). Sheared DNA was purified by solid-phase reversible immobilisation ( Strickland *et al.*, 2023): in brief, the method employs a 1.8X ratio of AMPure PB beads to sample to eliminate shorter fragments and concentrate the DNA. The concentration of the sheared and purified DNA was assessed using a Nanodrop spectrophotometer, Qubit Fluorometer and Qubit dsDNA High Sensitivity Assay kit. Fragment size distribution was evaluated by running the sample on the FemtoPulse system.

RNA was extracted from abdomen tissue of ilMytVite1 in the Tree of Life Laboratory at the WSI using the RNA Extraction: Automated MagMax™ *mir*Vana protocol ( do Amaral *et al.*, 2023). The RNA concentration was assessed using a Nanodrop spectrophotometer and a Qubit Fluorometer using the Qubit RNA Broad-Range Assay kit. Analysis of the integrity of the RNA was done using the Agilent RNA 6000 Pico Kit and Eukaryotic Total RNA assay.

Protocols developed by the WSI Tree of Life laboratory are publicly available on protocols.io ( Denton *et al.*, 2023b).

### Sequencing

Pacific Biosciences HiFi circular consensus DNA sequencing libraries were constructed according to the manufacturers’ instructions. Poly(A) RNA-Seq libraries were constructed using the NEB Ultra II RNA Library Prep kit. DNA and RNA sequencing was performed by the Scientific Operations core at the WSI on Pacific Biosciences Sequel IIe (HiFi) and Illumina NovaSeq 6000 (RNA-Seq) instruments. Hi-C data were also generated from head and thorax tissue of ilMytVite1 using the Arima v2 kit. The Hi-C sequencing was performed using paired-end sequencing with a read length of 150 bp on the Illumina NovaSeq 6000 instrument.

### Genome assembly and curation

Assembly was carried out with Hifiasm ( Cheng *et al.*, 2021) and haplotypic duplication was identified and removed with purge_dups ( Guan *et al.*, 2020). The assembly was then scaffolded with Hi-C data ( Rao *et al.*, 2014) using YaHS ( Zhou *et al.*, 2023). The assembly was checked for contamination and corrected as described previously ( Howe *et al.*, 2021). Manual curation was performed using HiGlass ( Kerpedjiev *et al.*, 2018) and PretextView ( Harry, 2022). The mitochondrial genome was assembled using MitoHiFi ( Uliano-Silva *et al.*, 2023), which runs MitoFinder ( Allio *et al.*, 2020) or MITOS ( Bernt *et al.*, 2013) and uses these annotations to select the final mitochondrial contig and to ensure the general quality of the sequence.

### Evaluation of final assembly

A Hi-C map for the final assembly was produced using bwa-mem2 ( Vasimuddin *et al.*, 2019) in the Cooler file format ( Abdennur & Mirny, 2020). To assess the assembly metrics, the *k*-mer completeness and QV consensus quality values were calculated in Merqury ( Rhie *et al.*, 2020). This work was done using Nextflow ( Di Tommaso *et al.*, 2017) DSL2 pipelines “sanger-tol/readmapping” ( Surana *et al.*, 2023a) and “sanger-tol/genomenote” ( Surana *et al.*, 2023b). The genome was analysed within the BlobToolKit environment ( Challis *et al.*, 2020) and BUSCO scores ( Manni *et al.*, 2021; Simão *et al.*, 2015) were calculated.

Table 3 contains a list of relevant software tool versions and sources.

**Table 3.** Software tools: versions and sources.

Software tool	Version	Source
BlobToolKit	4.1.7	https://github.com/blobtoolkit/blobtoolkit
BUSCO	5.3.2	https://gitlab.com/ezlab/busco
Hifiasm	0.16.1-r375	https://github.com/chhylp123/hifiasm
HiGlass	1.11.6	https://github.com/higlass/higlass
Merqury	MerquryFK	https://github.com/thegenemyers/MERQURY.FK
MitoHiFi	2	https://github.com/marcelauliano/MitoHiFi
PretextView	0.2	https://github.com/wtsi-hpag/PretextView
purge_dups	1.2.3	https://github.com/dfguan/purge_dups
sanger-tol/genomenote	v1.0	https://github.com/sanger-tol/genomenote
sanger-tol/readmapping	1.1.0	https://github.com/sanger-tol/readmapping/tree/1.1.0
YaHS	1.2a	https://github.com/c-zhou/yahs

### Genome annotation

The BRAKER2 pipeline ( Brůna *et al.*, 2021) was used in the default protein mode to generate annotation for the *Mythimna vitellina* assembly (GCA_949316375.1) in Ensembl Rapid Release at the EBI.

### Wellcome Sanger Institute – Legal and Governance

The materials that have contributed to this genome note have been supplied by a Darwin Tree of Life Partner. The submission of materials by a Darwin Tree of Life Partner is subject to the **‘Darwin Tree of Life Project Sampling Code of Practice’**, which can be found in full on the Darwin Tree of Life website here. By agreeing with and signing up to the Sampling Code of Practice, the Darwin Tree of Life Partner agrees they will meet the legal and ethical requirements and standards set out within this document in respect of all samples acquired for, and supplied to, the Darwin Tree of Life Project.

Further, the Wellcome Sanger Institute employs a process whereby due diligence is carried out proportionate to the nature of the materials themselves, and the circumstances under which they have been/are to be collected and provided for use. The purpose of this is to address and mitigate any potential legal and/or ethical implications of receipt and use of the materials as part of the research project, and to ensure that in doing so we align with best practice wherever possible. The overarching areas of consideration are:

•   Ethical review of provenance and sourcing of the material

•   Legality of collection, transfer and use (national and international)

Each transfer of samples is further undertaken according to a Research Collaboration Agreement or Material Transfer Agreement entered into by the Darwin Tree of Life Partner, Genome Research Limited (operating as the Wellcome Sanger Institute), and in some circumstances other Darwin Tree of Life collaborators.

## Data Availability

European Nucleotide Archive: *Mythimna vitellina*. Accession number PRJEB58960; https://identifiers.org/ena.embl/PRJEB58960 ( Wellcome Sanger Institute, 2023). The genome sequence is released openly for reuse. The *Mythimna vitellina* genome sequencing initiative is part of the Darwin Tree of Life (DToL) project. All raw sequence data and the assembly have been deposited in INSDC databases. Raw data and assembly accession identifiers are reported in Table 1.

## Author information

Members of the Natural History Museum Genome Acquisition Lab are listed here: https://doi.org/10.5281/zenodo.7139035.

Members of the Darwin Tree of Life Barcoding collective are listed here: https://doi.org/10.5281/zenodo.4893703.

Members of the Wellcome Sanger Institute Tree of Life Management, Samples and Laboratory team are listed here: https://doi.org/10.5281/zenodo.10066175.

Members of Wellcome Sanger Institute Scientific Operations: Sequencing Operations are listed here: https://doi.org/10.5281/zenodo.10043364.

Members of the Wellcome Sanger Institute Tree of Life Core Informatics team are listed here: https://doi.org/10.5281/zenodo.10066637.

Members of the Tree of Life Core Informatics collective are listed here: https://doi.org/10.5281/zenodo.5013541.

Members of the Darwin Tree of Life Consortium are listed here: https://doi.org/10.5281/zenodo.4783558.
