# Supplementary material for: The Genome Sequence of the Delicate moth, Mythimna vitellina (Hübner, [1808])
Source: Wellcome Open Res. 2024 Jul 29;9:416. [Version 1] doi: 10.12688/wellcomeopenres.22620.1 (PMC11599806; doi:10.12688/wellcomeopenres.22620.1)
Supplement: Peer review history

# Reviewer report: Reviewer response for version 1

**DOI:** 10.21956/wellcomeopenres.24920.r100925

**Published:** 2024-11-26

**Authors:** Annabel Whibley

**Affiliations:** 1 The University of Auckland, Auckland, Auckland, New Zealand; 2 Grapevine Improvement, Bragato Research Institute, Lincoln, New Zealand

In this Data Note, Sterling, Lees and colleagues report the genome assembly and annotation of the Delicate moth, *Mythimna vitellina.*

 The genome assembly appears to be of exceptionally high quality, with excellent completeness, contiguity and accuracy statistics. The HiC contact map and Blobplot are both very clean visualisations and attest to the assembly quality. Annotation has been performed with RNA evidence via the Ensembl pipeline. Methods are  well described and appropriate, sample and analysis metadata is carefully recorded and the links to sequence and assembly accessions are active.

Minor comments (both in background section)

 “… with a wingspan of 36–43 mm in the UK”  I found this statement a little confusing. Presumably this range is somewhat different from that found over the full species range, but it might be helpful to cite the UK stats within the global reported range, if that’s possible to do.

 I also don’t fully follow the logic of the opening background paragraph- which starts with forewing pattern, then hindwing and then returns to forewing.  Could/Should  this statement come earlier in the section and/or replace some of the earlier forewing commentary? “M. vitellina is a distinctive species with a characteristic wing pattern and yellow to brick coloured forewings (the derivation of vitellina is vitellus, which is Latin for egg yolk), both of which are unusual for Wainscot moths.”

Are sufficient details of methods and materials provided to allow replication by others?

Yes

Is the rationale for creating the dataset(s) clearly described?

Yes

Are the datasets clearly presented in a useable and accessible format?

Yes

Are the protocols appropriate and is the work technically sound?

Yes

Reviewer Expertise:

Genomics, Bioinformatics

I confirm that I have read this submission and believe that I have an appropriate level of expertise to confirm that it is of an acceptable scientific standard.

# Reviewer report: Reviewer response for version 1

**DOI:** 10.21956/wellcomeopenres.24920.r110800

**Published:** 2024-11-13

**Authors:** Bhagya C Thimmappa

**Affiliations:** 1 USDA, USDA Agricultural Research Service, Chatsworth, New Jersey, USA

The authors of a paper titled " **The Genome Sequence of the Delicate moth, *Mythimna vitellina* (Hübner, [1808])**" report a chromosomal-level genome assembly for male *Mythimna vitellina *along with genome annotation and mitochondrial genome assembly. The assembly spans 726.7 megabases and is nearly complete, with a busco value of 98.8% using the lepidoptera_odb10 dataset.

 The paper reads well, with enough introduction and a well-described method section. The data set reported here will be a great resource for understanding moth evolution and how they differ from their closely related organisms.

 Minor comments:

 1. For a broader audience, please explain the term "noctuid" in the background section.

 2. It is mentioned that " *M. vitellina* is a distinctive species with a characteristic wing pattern." Would it be possible to get a better image where the wing pattern is highlighted/mentioned?

Are sufficient details of methods and materials provided to allow replication by others?

Yes

Is the rationale for creating the dataset(s) clearly described?

Yes

Are the datasets clearly presented in a useable and accessible format?

Yes

Are the protocols appropriate and is the work technically sound?

Yes

Reviewer Expertise:

Bioinformatics, Omics, High-Throughput Sequencing and Analysis

I confirm that I have read this submission and believe that I have an appropriate level of expertise to confirm that it is of an acceptable scientific standard.
